# Timing of Intubation in Covid-19 ARDS: What “time” really matters?

**DOI:** 10.1186/s13054-021-03598-2

**Published:** 2021-05-21

**Authors:** Vasiliki Tsolaki, George E. Zakynthinos

**Affiliations:** Critical Care Department, University Hospital of Larissa, University of Thessaly, Faculty of Medicine, Mezourlo, 41110 Larissa, Greece

**To the Editor,**

We congratulate Dr. Papoutsi et al. for their study, on the optimal time of intubation in Covid-19 ARDS patients [1]. In their systematic meta-analysis they found that outcomes are not affected from the intubation time (within 24-h of ICU admission or later).

We agree with the authors that “early intubation”, relying solely on the time spent in the Intensive Care Unit (ICU) before intubation, is a rather arbitrary definition and may be misleading. Many important data are lost in this type of analysis, which may affect outcomes. Firstly, ARDS severity in patients receiving early vs late intubation is not reported. Were patients in the two groups of different severity? The authors acknowledge this limitation, but disease severity (as defined by the oxygenation impairment, lung infiltration extent, APACHE II score) and patient heterogeneity, arising from differences in clinical practices, impacts outcomes. Probably, these criteria may be more meaningful than just “time”.

Secondly, ICU admission criteria vary according to resource availability and institution protocols. Thus, hospitalization of non-intubated patients in ICUs is not widely applicable. A substantial proportion of information regarding the optimal intubation time is carried in patients intubated in the wards, and this group has not been included in the analysis. Probably, the time the patients have spent under “distress” during hospitalization, should not be neglected. Therefore, other variables, besides frank “time”, should be sought to decide when to intubate. In our opinion, the cumulative time with hypoxemia and/or tachypnea are meaningful data to look at. In other words, is Patient-Self Inflicted Lung Injury a matter to care for in Covid-19 ARDS, or should we tolerate hypoxemia and tachypnea to minimize complications from sedation and mechanical ventilation [2, 3]? It would be very informative to know whether early intubation preserves respiratory mechanics.

Optimal intubation time is an issue of great importance, either performed in the ICU or elsewhere, and should be investigated in future, carefully-designed trials. “Silent hypoxia” was one of the initial observations in Covid-19 pathophysiology, present despite extensive pulmonary infiltrates and severe hypoxemia [4]; prone position has been widely adopted in sedated and non-sedated patients, altering oxygenation status and therefore intubation time [5].

The decision to intubate may be an “art of medicine”, yet, in times of such crisis, when doctors from different fields and with different skills, or even young doctors without specialties, are encountered in the decision making, formal thresholds and sound protocols should be introduced.

## Data Availability

Not applicable.
