# Supplementary material for: Timing of Intubation in Covid-19 ARDS: What “time” really matters?
Source: Crit Care. 2021 May 21;25:173. doi: 10.1186/s13054-021-03598-2 (PMC8139220; doi:10.1186/s13054-021-03598-2)
Supplement: Related texts

# Authors’ reply

**Authors:** E. Papoutsi, V. G. Giannakoulis, E. Xourgia, C. Routsi, A. Kotanidou, I. I. Siempos

Dear Editor-in-Chief,

We thank Drs Tsolaki and Zakynthinos for their interest in our work [1] and insightful points.

We agree with our colleagues that defining “early” intubation solely based on a time threshold spent in the intensive care unit (ICU) might be arbitrary. We accordingly explored an alternate definition of “early” intubation, using as criterion a prior trial of high flow nasal cannula (HFNC) or non-invasive mechanical ventilation (NIV). This sensitivity analysis aimed to address the notion that patients maintained in spontaneous breathing (HFNC) or receiving partial ventilatory assistance (NIV) may experience high respiratory drive leading to high tidal volume and transpulmonary pressure, which may in turn contribute to development of patient self-inflicted lung injury (P-SILI) [3]. That being said, one should keep in mind that scientific evidence regarding the clinical relevance of P-SILI in humans may not yet be compelling enough to justify early intubation [6].

Also, our colleagues reasonably pointed out that criteria for ICU admission may vary depending on institution protocols and availability of resources. Indeed, availability of resources may substantially influence not only overall outcomes [7] but also the threshold of ICU admission [8]. We attempted to address this issue by performing a subgroup analysis of reports from regions with low disease burden (namely, Germany, Korea and Greece) and we again found that all-cause mortality remained comparable between the two (“early” versus “late” intubation) groups.

Overall, “early” intubation represents a spectrum of definitions. Our meta-analysis explored two (one with and one without a time threshold) and revealed no benefit of early intubation. Therefore, we attempted to convey the message that following a stepwise approach to intubation, by offering the patient a trial of HFNC or NIV, seems reasonable based on the current evidence. Practicing medicine is a cost–benefit balance and it seems that preemptive intubation in critically ill patients with COVID-19 is becoming increasingly difficult to justify.
